# A Positive Feedback Loop Between DICER1 and Differentiation Transcription Factors Is Important for Thyroid Tumorigenesis

**DOI:** 10.1089/thy.2020.0297

**Published:** 2021-06-08

**Authors:** Julia Ramírez-Moya, Pilar Santisteban

**Affiliations:** ^1^ Instituto de Investigaciones Biomédicas “Alberto Sols,” Consejo Superior Investigaciones Científicas, and Universidad Autónoma de Madrid (CSIC-UAM), Madrid, Spain.; ^2^ Centro de Investigación Biomédica en Red de Cáncer (CIBERONC), Instituto de Salud Carlos III (ISCIII), Madrid, Spain.

**Keywords:** thyroid transcription factors, CREB, tumor suppressor DICER1, thyroid cancer

## Abstract

***Background:*** DICER1 plays a central role in microRNA biogenesis and functions as a tumor suppressor in thyroid cancer, which is the most frequent endocrine malignancy with a rapidly increasing incidence. Thyroid cancer progression is associated with loss of cell differentiation and reduced expression of thyroid differentiation genes and response to thyrotropin (TSH). Here we investigated whether a molecular link exists between DICER1 and thyroid differentiation pathways.

***Methods:*** We used bioinformatic tools to search for transcription factor binding sites in the *DICER1* promoter. DICER1, NKX2-1, PAX8, and CREB expression levels were evaluated by gene and protein expression *in vitro* and by interrogation of The Cancer Genome Atlas (TCGA) thyroid cancer data. Transcription factor binding and activity were assayed by chromatin immunoprecipitation, band-shift analysis, and promoter–reporter gene activity. Gene-silencing and overexpression approaches were used to elucidate the functional link between DICER1 and differentiation.

***Results:*** We identified binding sites for NKX2-1 and CREB within the *DICER1* promoter and found that both transcription factors are functional in thyroid cells. TSH induced *DICER1* expression in differentiated thyroid cells, at least in part, through the cAMP/PKA/CREB pathway. TCGA analysis revealed a significant positive correlation between CREB and DICER1 expression in human thyroid tumors. NKX2-1 overexpression increased *DICER1* promoter activity and expression *in vitro*, and this was significantly greater in the presence of CREB and/or PAX8. Gain- and loss-of-function assays revealed that DICER1 regulates NKX2-1 expression in thyroid tumor cells and *vice versa*, thus establishing a positive feedback loop between both proteins. We also found a positive correlation between NKX2-1 and DICER1 expression in human thyroid tumors. DICER1 silencing decreased PAX8 expression and, importantly, the expression and activity of the sodium iodide symporter, which is essential for the diagnostic and therapeutic use of radioiodine in thyroid cancer.

***Conclusions*:** The differentiation transcription factors NKX2.1, PAX8, and CREB act in a positive feedback loop with DICER1. As the expression of these transcription factors is markedly diminished in thyroid cancer, our findings suggest that DICER1 downregulation in this cancer is mediated, at least partly, through impairment of its transcription.

## Introduction

The highly conserved RNAse III enzyme DICER1 is crucial for the biogenesis of microRNAs (miRNAs) by cleaving double-stranded small-noncoding RNA precursors to generate mature miRNAs of 21–24 nucleotides. Several studies have highlighted an association between the reduced expression of DICER1 and poor prognosis in different cancers, including lung, breast, skin, endometrial, and ovarian cancer (1–5). We recently showed that DICER1 acts as a tumor suppressor in thyroid cancer, as its downregulation promotes proliferation, migration, and invasion (4). We also found that low *DICER1* levels in thyroid carcinomas correlate with a worse clinical outcome (3).

Thyroid cancer is the most frequent endocrine malignancy whose incidence has increased (6). Well-differentiated papillary thyroid cancer (PTC) and follicular thyroid cancer (FTC) account for most cases and usually have a good prognosis. By contrast, undifferentiated anaplastic thyroid cancer (ATC) is an extremely aggressive cancer with a five-year disease-free survival rate of almost 0%. The classical view of thyroid cancer pathogenesis considers thyroid carcinomas as tumors that progress through a dedifferentiation process, which underscores the need to identify therapeutic targets that drive thyroid cancer differentiation (7).

Thyroid differentiation is finely tuned by positive and negative regulatory signals (8,9). Thyrotropin (TSH) engages its cognate G protein-coupled receptor, TSH receptor (TSHR), to activate cAMP/PKA/CREB signaling, which regulates the transcription of thyroid-specific genes such as thyroglobulin, thyroperoxidase, and sodium iodide symporter (NIS) (10,11). In turn, these genes are regulated by the instructive transcription factors NKX2-1, PAX8, and FOXE1, which define the differentiated thyroid phenotype (12,13).

TSHR signaling is required for thyroid carcinogenesis in a mouse model of spontaneous FTC (14). In addition, dedifferentiated thyroid tumors lose their response to TSH and its downstream signaling (15,16). Importantly, thyroid cancer shows a marked downregulation of differentiation, and consequently, a characteristic decrease in the expression of thyroid transcription factors is critical for cancer progression (11,13).

Normal developmental processes and cancer share many pathways related to cell proliferation and differentiation. In this respect, conditional deletion of *Dicer1* in thyroid follicular cells during mouse development causes a strong reduction in the expression of differentiation markers such as *Nkx2-1* and *Pax8* (17) and *NIS* later in adults (18). Moreover, mice are hypothyroid at birth and present characteristics of neoplastic alterations in thyroid tissue in adult life (17). These findings suggest that miRNAs are necessary to maintain thyroid tissue homeostasis.

Given the crucial role of DICER1 in thyroid cancer aggressiveness, the present study was designed to uncover the transcriptional mechanisms that control its expression. We also explored whether DICER1 downregulation is involved in differentiation by regulating the instructional transcription factors involved in this process.

## Methods

### Bioinformatic predictions

Transcription factor binding sites within the human *DICER1* promoter were identified using the ECR browser. The cBioPortal for Cancer Genomics was used to obtain the correlations between *DICER1*, *CREB*, and *CREM* mRNA levels, on the thyroid carcinoma data set of *The Cancer Genome Atlas* (TCGA) database.

### Patients

Paired samples of PTC tumors and contralateral normal thyroid tissue from patients (*n* = 7) were studied as previously described (19). Written informed consent was obtained from all the patients following the protocols approved by the Hospital ethics committee.

### Cell culture and transfection

Cells, culture methods, and transfection (20) are described in Supplementary Data.

### Luciferase assay

The pGL3-DICER-Prom vector or serial deletion mutants were transfected together with pCMV-*Renilla* and the corresponding transcription factor expression vectors. Promoter activity was assayed as described previously (9).

### RNA quantification, protein extraction, and Western blotting

RNA quantification, protein extraction methods, and Western blotting are described in Supplementary Data. The primers used are described in Supplementary Table S1.

### DNA-NKX2.1 binding assays

Chromatin immunoprecipitation (ChIP) and electrophoretic mobility shift assay (EMSA) details are described in Supplementary Data.

### Iodide transport

PCCl3 cells were assayed for iodide transport as previously described (21), and are detailed in the Supplementary Data.

### Statistical analysis

Results are expressed as the mean ± standard deviation of at least three different experiments performed in triplicate. Statistical significance was determined by Student's *t*-test (two-tailed) analysis and differences were considered significant at a *p*-value <0.05.

## Results

### DICER1 promoter contains CRE motifs and binding sites for NKX2-1

**FIG. 1.**
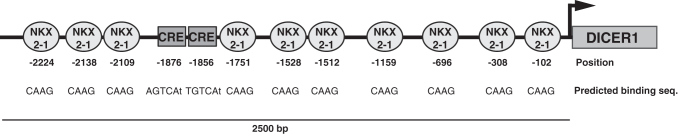
Schematic representation of the promoter region of human *DICER1* showing predicted NKX2-1 binding sites and CRE motifs. The relative position from the transcriptional start site (+1) is reported below each transcription factor binding site. Also, the CRE and NKX2-1 consensus sequences are listed below each putative binding site.

DICER1 acts as a novel tumor suppressor and is downregulated in thyroid cancer (3); however, the mechanism of this downregulation and its transcriptional regulation in the thyroid gland is unknown. We conducted *in silico* analysis of consensus transcription factor binding sites in the human *DICER1* promoter, considering 2500 base pairs upstream of the transcription initiation site. We identified two canonical CRE motifs at positions −1876 (consensus sequence AGTCAt) and −1856 (consensus sequence TGTCAt) and 10 NKX2-1 consensus binding sites (CAAG) at −102, −308, −696, −1159, −1512, −1528, −1751, −2109, −2138, and −2224 (Fig. 1). Of note, CREB, the main transcription factor that binds to CRE elements, and NKX2-1 have fundamental roles in thyroid differentiation (13), and their loss is a hallmark of thyroid cancer progression. Accordingly, the decrease in *DICER1* levels in patients with thyroid cancer could be explained by the dedifferentiation events occurring in tumors. For these reasons, we examined the roles of these transcription factors in DICER1 regulation.

### TSH regulates DICER1 through the cAMP/CREB-signaling pathway in thyroid cells

The finding of CRE consensus sequences in the *DICER1* promoter led us to hypothesize that TSH, through TSHR/cAMP signaling, activates *DICER1* to increase DICER1 protein levels. Undifferentiated thyroid tumors lose their response to TSH and its downstream signaling, and this loss in the most undifferentiated tumors could explain, at least in part, the DICER1 downregulation observed in thyroid tumors.

**FIG. 2.**
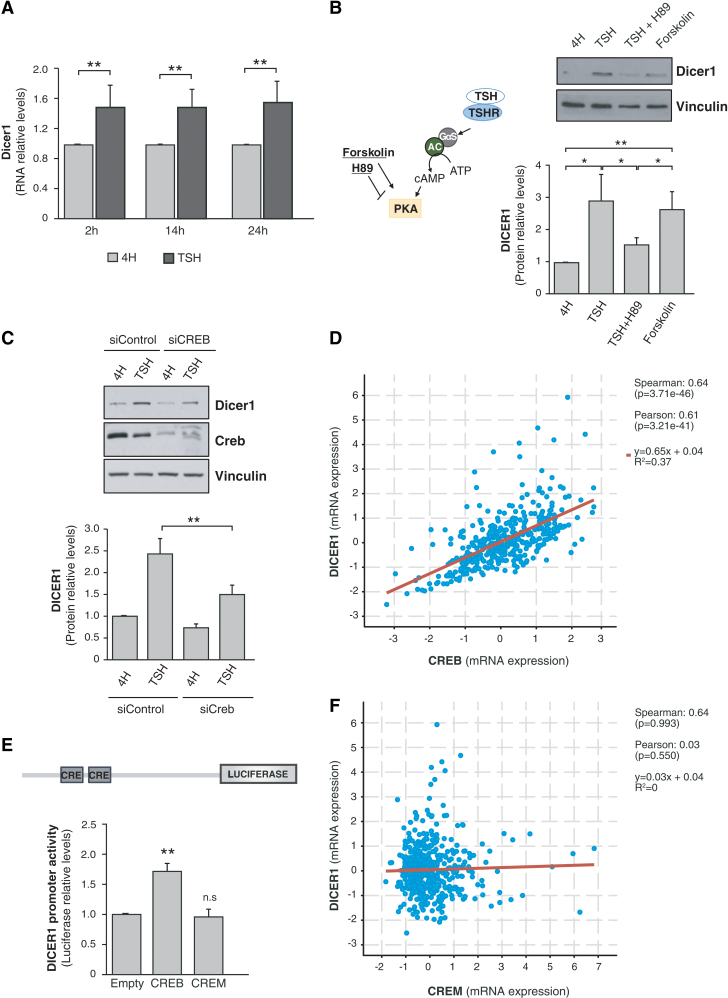
TSH and CREB induce *DICER1* promoter activity. (**A**) Relative mRNA levels of *Dicer1* in PCCl3 cells after TSH stimulation for 2, 14, or 24 hours. (**B**) Left: Schematic representation of the TSH pathway, showing the function of forskolin and the inhibitor H89. Right: Representative immunoblot (upper panel) and quantification (lower panel) for Dicer1. (**C**) PCCl3 cells were transfected with control (siControl) or CREB (siCREB) siRNAs. Cells were maintained for 48 hours in starvation medium (4H) after transfection and then treated with TSH for 24 hours. Upper panel: Representative immunoblot of Dicer1. Lower panel: Dicer1 quantification. Vinculin was used as loading control in B and C panels. (**D**) Correlation analysis of *DICER1* and *CREB* in thyroid cancer patients from the TCGA database. Statistics are shown on the right. (**E**) Effect of CREB and CREM on *DICER1* promoter activity in HeLa cells evaluated as luciferase activity relative to *Renilla* levels 72 hours after cotransfection with pGL3-DICER-Prom and CREB or CREM expression vectors. (**F**) Correlation analysis of *DICER1* and *CREM* in thyroid cancer patients from TCGA database. Statistics are shown on the right. Values represent mean + SD (*n* = 3). *p < 0.05; **p < 0.01. ns, nonsignificant; SD, standard deviation; TCGA, The Cancer Genome Atlas; TSH, thyrotropin. Color images are available online.

We first studied the regulation of *Dicer1* by TSH/cAMP in the TSH-responsive differentiated rat thyroid cell line PCCl3 (22). Cells were starved and maintained in 4H medium for 48 hours and then stimulated with TSH for 2, 14, or 24 hours. We observed that TSH significantly increased *Dicer1* mRNA levels at the three time points studied (Fig. 2A). This effect was mimicked by forskolin and inhibited by H89 (Fig. 2B), suggesting that TSH induces Dicer1 mRNA and protein levels *via* cAMP/PKA signaling. As the CREB transcription factor is a major final effector of cAMP/PKA signaling, we assessed its involvement in TSH-induced *DICER1* expression by silencing its expression in PCCl3 cells. As shown in Figure 2C, inhibiting CREB expression significantly reduced TSH induction of DICER1. These results indicate that TSH-dependent stimulation of *DICER1* expression is mediated, at least partly, by CREB. Supporting this, an analysis of the mRNA levels of *CREB* and *DICER1* in 496 human tumors from the TCGA database revealed a significant positive correlation between the two (Fig. 2D). Indeed, both *CREB* and *DICER1* were downregulated in PTC samples relative to normal samples, with a fold change of 0.87 and 0.56, respectively (Supplementary Fig. S1A) (3).

We next analyzed the transcriptional control of *DICER1* by CREB, finding that CREB overexpression activated a human *DICER1* promoter construct (Fig. 2E). By contrast, the overexpression of CREM, a transcription factor that also binds CRE elements present in the promoters of cAMP-regulated genes, failed to activate the promoter (Fig. 2E). This latter observation is in good agreement with the lack of correlation between *CREM* and *DICER1* mRNA levels in human thyroid tumors (Fig. 2F).

### Existence of a feedback loop between NKX2-1 and DICER1

**FIG. 3.**
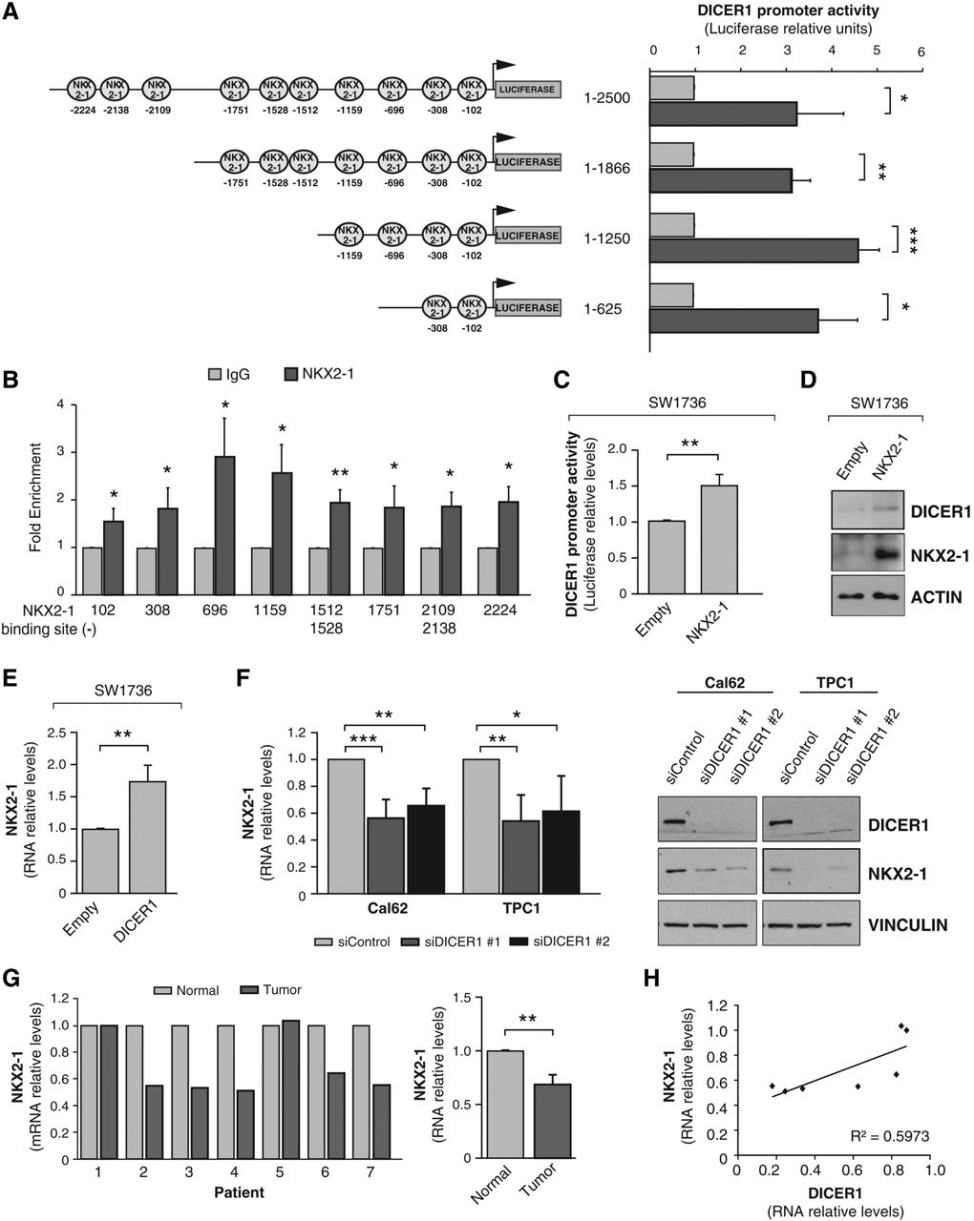
NKX2-1 increases *DICER1* promoter activity and DICER1 induces and correlates with NKX2-1 levels. (**A**) Left panel: schematic representation of the cloned human *DICER1* promoter fragments. Circles indicate the putative NKX2-1 binding sites identified *in silico*. The relative position from the transcriptional start site (+1) is reported below each transcription factor binding site. Right panel: HeLa cells were cotransfected with the indicated promoter-luciferase construct and the NKX2-1 expression vector. Luciferase activity relative to *Renilla* levels was assayed 72 hours after cotransfection. (**B**) ChIP analysis of NKX2-1 binding to *DICER1* promoter. qPCR analysis of ChIP performed on BCPAP cells with NKX2-1 antibody or IgG as a control. Numbers below represent the NKX2-1 predicted sites in the *DICER1* promoter. (**C**) NKX2-1 induces *DICER1* promoter activity in SW1736 thyroid cancer cells, evaluated as luciferase activity relative to *Renilla* levels 72 hours after cotransfection with pGL3-DICER-Prom and an NKX2.1 expression vector. (**D**) Immunoblot for DICER1 and NKX2-1 in SW1736 cells 48 hours after transfection with the NKX2-1 expression vector or an empty vector. ACTIN was used as loading control. (**E**) *NKX2-1* mRNA levels in SW1736 cells 48 hours after DICER1 transfection. (**F**) The thyroid cancer Cal62 and TPC1 cell lines were transfected with two siRNAs against DICER1 (siDICER1 #1 and siDICER1 #2) or a control siRNA (siControl), and *NKX2-1* mRNA levels (left panel) and protein levels (right panel) were measured 72 hours after transfection. (**G**) Left: relative *NKX2-1* mRNA expression levels in seven patients with PTC (contralateral and normal thyroid tissue). Right: total average of *NKX2-1* mRNA relative levels. (**H**) Correlation analysis of *DICER1* and *NKX2-1* in seven patients with PTC. *DICER1* levels were analyzed in Ramirez-Moya *et al.* (3). Values represent mean + SD (*n* = 3). *p < 0.05; **p < 0.01; ***p < 0.001. ChIP, chromatin immunoprecipitation; PTC, papillary thyroid cancer; qPCR, quantitative polymerase chain reaction.

*In silico* analysis for thyroid transcription factor binding sites in the human *DICER1* proximal promoter predicted several NKX2-1 consensus binding sites (Fig. 1). The transcription factors NKX2-1, PAX8, and FOXE1 are key developmental genes that define the differentiated phenotype of thyroid cells. As cell differentiation is disrupted along thyroid cancer progression and DICER1 is downregulated in thyroid cancer, we next examined the possible transcriptional regulation of *DICER1* by NKX2-1 by assessing the functionality of NKX2-1 over the *DICER1* promoter. We found that transfected NKX2-1 activated the whole −2500 bp *DICER1* promoter in HeLa cells (Fig. 3A). To determine the more critical *DICER1* promoter regions for NKX2-1-mediated activation, we made serial deletions of the *DICER1* promoter (−1866 − 1250, and −625 bp), finding that the greatest luciferase activity occurred in the p*DICER*-1250-Luc construct containing the four predicted NKX2-1 binding sites closest to the transcription start site (Fig. 3A), suggesting that the proximal region is responsible for NKX2-1 transcriptional activity. We validated this using ChIP quantitative polymerase chain reaction assays with specific primers to amplify the different NKX2-1 binding sites within the *DICER1* promoter. As shown in Figure 3B, NKX2-1 bound to all the predicted sites, with the maximum fold enrichment at sites located at positions −696 and −1159. The specific NKX2-1 binding at position −696 was confirmed by EMSA (Supplementary Fig. S1B).

NKX2-1 activation of the *DICER1* promoter was also reproduced in the ATC cell line SW1736 (Fig. 3C). In addition, overexpression of NKX2-1 increased DICER1 protein levels in SW1736 cells (Fig. 3D), overall confirming the functionality of the NKX2-1 binding sites in the *DICER1* promoter, and pointing to a role for NKX2-1 in *DICER1* transcription.

We next performed the opposite experiment and overexpressed DICER1 in SW1736 cells, finding a significant increase in *NKX2-1* mRNA levels (Fig. 3E). As expected, DICER1 silencing in the ATC lines Cal62 and TPC1 using two different siRNAs significantly decreased *NKX2-1* mRNA and protein levels compared with nonsilenced cells (Fig. 3F). These data suggest a role for DICER1 in thyroid differentiation and the existence of a positive feedback loop between DICER1 and NKX2-1.

We confirmed some of these *in vitro* findings in human thyroid tumors. We observed that *NKX2-1* expression was lower in thyroid tumor tissue than in paired normal tissue (Fig. 3G), which is consistent with the described loss of differentiation. We previously described low levels of *DICER1* in the same tumors (3). Analysis of *DICER1* expression values together with those described here for *NKX2-1* revealed a positive correlation (*R*^2^ = 0.5973) between both genes (Fig. 3H).

### PAX8 cooperates with NKX2-1 and CREB to regulate DICER1

**FIG. 4.**
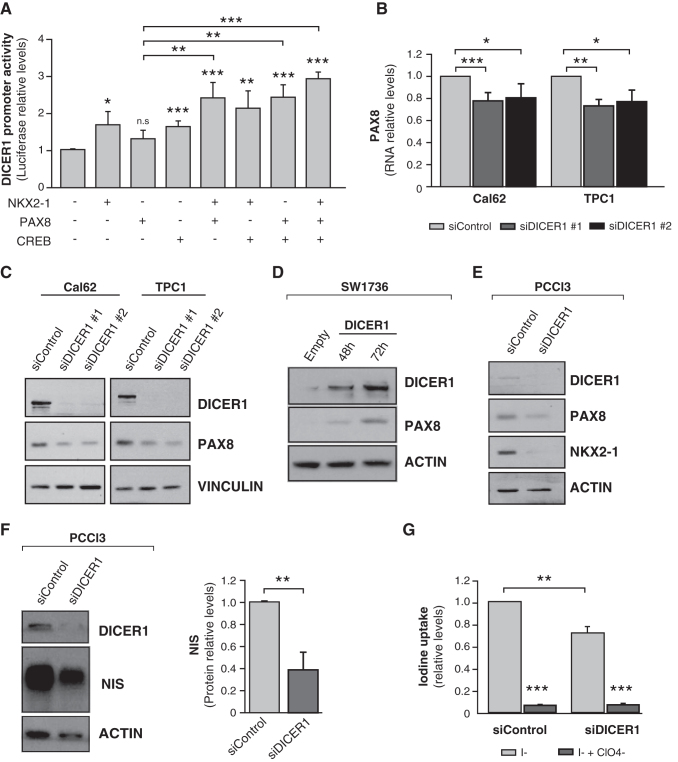
PAX8 cooperates to induce *DICER1* transcription, which in turn increases PAX8 and NIS expression. (**A**) HeLa cells were assayed for luciferase activity 72 hours after transfection of pGL3-DICER-Prom (*DICER1* promoter) and the expression vectors NKX2-1, CREB, PAX8, alone or in combination. (**B, C**) The thyroid cancer Cal62 and TPC1 cell lines were transfected with two siRNAs against DICER1 (siDICER1 #1 and siDICER1 #2) or a control siRNA (siControl), and *PAX8* mRNA (**B**) or protein (**C**) levels were measured. (**D**) Representative immunoblot for PAX8 and DICER1 in SW1736 cells overexpressing DICER1 at the indicated times. (**E, F**) PCCL3 cells were transfected with a DICER1 siRNA (siDICER1) or a control (siControl). Representative immunoblots for PAX8 and NKX2-1 **(E)** and NIS **(F)** are shown. NIS protein quantification is shown on the right panel. ACTIN was used as loading control in panels (**C–F**). (**G**) Iodine uptake in PCCL3 cells 48 hours after transfection with the abovementioned siRNAs. Values represent mean + SD (*n* = 3). *p < 0.05; **p < 0.01; ***p < 0.001. NIS, sodium iodide symporter; ns, nonsignificant.

PAX8 is considered the main transcription factor for thyroid cell differentiation (23). *In silico* analysis of PAX8 consensus binding sites failed to detect any in the *DICER1* proximal promoter, and PAX8 was unable to enhance *DICER1* promoter activity in overexpression experiments in HeLa cells (Fig. 4A). Nevertheless, PAX8 cooperated with NKX2-1 and CREB, individually and together, to significantly increase *DICER1* promoter activity (Fig. 4A). These data reinforce the idea that in a differentiated state, regulated by TSH, the expression of all three transcription factors increases *DICER1* expression, whereas the loss of differentiation in thyroid cancer leads to the impairment of *DICER1* transcription, explaining the low levels of this thyroid tumor suppressor.

### DICER1 regulates the levels of the main differentiation transcription factors, leading to loss of NIS expression and iodine uptake

The positive feedback loop between DICER1 and NKX2-1 points to a role for DICER1 in thyroid differentiation. Two previous studies described that conditional deletion of *Dicer1* in thyroid follicular cells during mouse development leads to a strong reduction in the expression of Nkx2-1 and Pax8 (17), and in Nis in adult mice (18). We thus examined the link between DICER1 and thyroid differentiation markers. Silencing of DICER1 in Cal62 and TPC1 cell lines decreased *PAX8* mRNA (Fig. 4B) and protein (Fig. 4C) expression, whereas DICER1 overexpression had the opposite effect on PAX8 protein expression (Fig. 4D), confirming the involvement of DICER1 in PAX8 regulation.

We studied the expression of NKX2-1, PAX8, and DICER1 in the three thyroid cancer cell lines concurrently. We found a general concordance between the three (Supplementary Fig. S2), with the highest levels in the TPC1 papillary cell line, the most differentiated, supporting a regulatory network between these factors.

A major target of NKX2-1 and PAX8 is the NIS, which is the hallmark of thyroid differentiation. NIS expression and, by extension, function are necessary for the correct synthesis of thyroid hormone and for the diagnostic and therapeutic use of radioiodine in thyroid cancer (24). Using PCCl3 cells, we confirmed that silencing of Dicer1 resulted in a decrease in Nkx2-1 and Pax8 levels (Fig. 4E). NIS expression was significantly decreased in Dicer1-silenced PCCl3 cells (fold change 0.378) (Fig. 4F). This was concomitant with a loss of radioiodine uptake (Fig. 4G), overall suggesting a critical role for DICER1 in maintaining a differentiated thyroid phenotype.

## Discussion

Malignancy and differentiation status are inversely correlated. Thyroid cell differentiation is driven by the coordinated action of NKX2-1, PAX8, FOXE1, and HHEX transcription factors, which together regulate the thyroid-specific proteins responsible for thyroid hormone biosynthesis (12,13). Among these, the NIS is essential for treatment of thyroid cancer because radioiodine therapy depends on NIS expression and its correct location in the basolateral membrane of thyroid follicular cells (24,25). Likewise, the active response to TSH via TSHR/cAMP-mediated signaling in differentiated thyroid tumors is critical because TSH is used to induce radioactive iodine uptake (26,27).

Loss of thyroid-specific proteins and differentiation markers is common in thyroid carcinogenesis. Several studies have reported the impaired expression of thyroid transcription factors such as NKX2-1 and PAX8 in thyroid carcinomas, suggesting that their deregulation is pivotal for the initiation and progression of thyroid neoplasms (28–31). Interestingly, researchers at TCGA developed a thyroid differentiation score based on the expression of several genes, and found an inverse correlation between thyroid transcription factors and tumor malignancy (32).

We recently demonstrated that DICER1 downregulation in thyroid cancer cells correlates with an increase in proliferation, migration, and invasion (3). However, no study had examined the relationship between DICER1 and differentiation in thyroid cancer. The findings that DICER1 is related to thyroid differentiation during thyroid embryonic development (17,18), and that development and cancer share multiple processes, prompted us to analyze the regulation of *DICER1* by the main thyroid transcription factors, and the implication of DICER1 downregulation for dedifferentiation in thyroid cancer.

Bioinformatic analysis revealed ten NKX2-1 binding sites and two CREs within the *DICER1* promoter and functional analyses demonstrated that TSH via cAMP regulates the expression of *DICER1* in differentiated thyroid cells, an effect mediated by CREB. This might indicate that as the response to TSH is lost in tumors, the expression of CREB, and consequently that of DICER1, is also lost. This prediction is supported by the TCGA data, showing a downregulation of *DICER1* and *CREB* expression in tumors and a correlation between them.

We found that NKX2-1 induces *DICER1* promoter activity in heterologous assays in HeLa cells and, more importantly, in ATC cells (SW1736). Interestingly, inhibition analysis revealed that DICER1 is positively associated with NKX2-1 levels. The existence of a positive feedback loop between NKX2-1 and DICER1 might have important implications in thyroid cancer, as is supported by the positive correlation between both proteins in a series of patient samples analyzed here.

Akin to NKX2-1, PAX8 plays a key role in thyroid differentiation. However, neither the PAX8 consensus nor the degenerated sequence (33) was evident in the proximal *DICER1* promoter. While PAX8 by itself failed to activate *DICER1* transcription, it cooperated additively with both NKX2-1 and CREB, which is consistent with the observation of an interaction between NKX2-1 and PAX8 that synergistically activates transcription (34). In addition, CRE binding proteins interact with PAX8 to regulate NIS transcription (35,36). These data strongly suggest that the coordinated action of the three transcription factors, under the control of TSH, triggers *DICER1* expression in differentiated thyroid cells. In turn, loss of these differentiation factors reduces *DICER1* expression and results not only in dedifferentiation but also enhanced cell aggressiveness (3).

Prior studies showed that the absence of Dicer1 does not significantly affect the expression of Nkx2-1 and Pax8 at the end of embryonic development and at the beginning of birth (18); however, there is a decrease in both transcription factors in adults (17). These mice show severe hypothyroidism and loss of *Tshr*, *TPO*, and *Nis* expression with signs of neoplastic alterations.

Interestingly, among the cell lines studied, we observed that TPC1 cells have the highest levels of DICER1, PAX8, and NKX2-1. This fits with our hypothesis, as TPC1 is a papillary cell line and is therefore more differentiated than the anaplastic cell lines (Cal62 and SW1736). Also worthy of note is the finding that SW1736 cells show lower levels of DICER1, PAX8, and NKX2-1 than Cal62, suggesting that it is even less differentiated. This might be because SW1736 harbors mutations in BRAF^V600E^, whereas Cal62 harbors a mutation in KRAS^G12R^. The BRAF mutation is believed to confer higher aggressiveness and is associated with a lower differentiation score (32). Importantly, there is a concordance between the three factors in the three cell lines; the highest levels of PAX8 and NKX2-1 are found in cells with the highest levels of DICER1. These results support our hypothesis and reveal a regulatory network between the three factors.

We found that PAX8 and NKX2-1 levels are significantly reduced in *DICER1*-silenced human thyroid tumor cells, whereas the opposite is seen in DICER1 overexpressing cells. Similar results were obtained in *Dicer1-*silenced differentiated PCCl3 cells. Interestingly, the levels of Nis, the main target of Pax8, also decreased after *Dicer1* silencing, resulting in a significant reduction in iodine uptake. We previously reported low *DICER1* mRNA levels in thyroid tumors (3). This agrees with data from the Human Protein Atlas, in which DICER1 protein levels are decreased in thyroid tumor samples. Specifically, all control samples (from normal thyroid gland) show positive (although weak) staining in the majority (75%) of cells, whereas staining is weak or absent in thyroid carcinoma tissue and the fraction of stained cells is less than 25% in three of four samples. We speculate that the low DICER1 levels lead to differentiated tumors with a markedly decreased expression of NIS, making radioiodine treatment futile. Thus, the recovery of thyroid transcription factors should ultimately be effective for *DICER1* expression and for functional NIS activity.

**FIG. 5.**
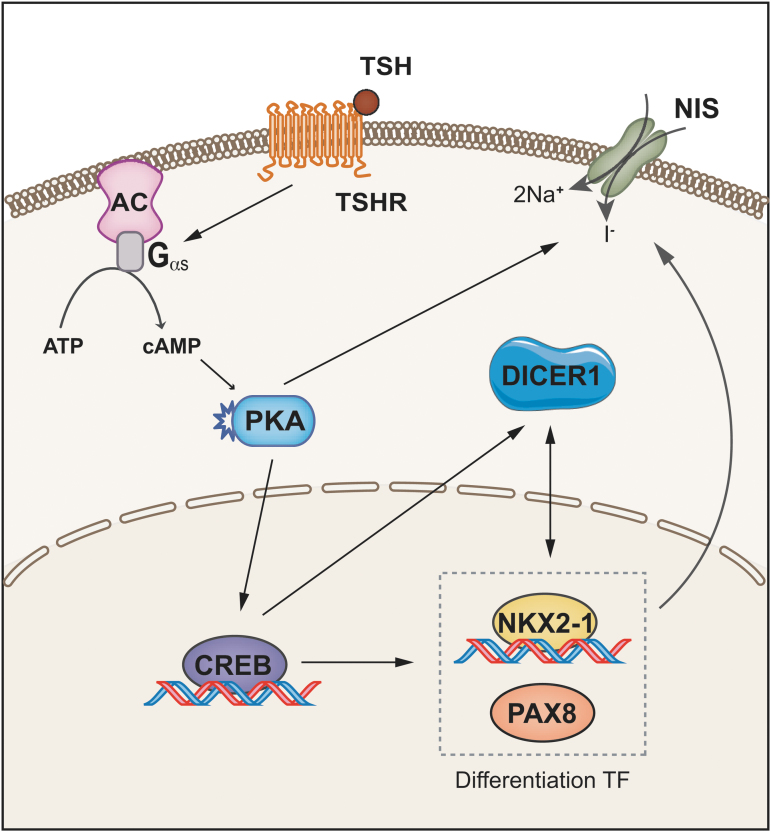
Schematic summary. TSH acting through its G protein-coupled receptor induces the cAMP/PKA/CREB pathway, which regulates the transcription of *DICER1*. A feedback loop exists between DICER1 and the thyroid transcription factors NKX2-1 and PAX8. Both transcription factors, in cooperation, induce the transcription of *DICER1*, which in turn controls PAX8 and NKX2-1 levels, inducing NIS expression and iodine uptake. TSHR, TSH receptor. Color images are available online.

In summary, we demonstrate that the miRNA biogenesis enzyme DICER1, acting as a tumor suppressor, is finely regulated in thyroid cells. Our results support the following scenario (Fig. 5): TSH, the main hormone controlling proliferation and differentiation in thyroid follicular cells, stimulates *DICER1* expression via TSHR/cAMP/PKA, CREB, NKX2-1, and PAX8 to cooperatively regulate DICER1, and a positive feedback loop between NKX2-1 and DICER1 implicates a role for DICER1 in thyroid differentiation. Indeed, DICER1 upregulates NKX2-1 and PAX8 levels inducing NIS expression and function, thereby increasing iodine uptake. Most of our findings were supported by an exhaustive analysis of TCGA and human tumoral samples, allowing us to conclude that DICER1 downregulation in thyroid tumors is mediated, at least partly, through the impairment of its transcription.

## Supplementary Material

Supplemental data

Supplemental data

Supplemental data

Supplemental data

## References

[1] Lin S, Gregory RI 2015 MicroRNA biogenesis pathways in cancer. Nat Rev Cancer 15:321–333 25998712 10.1038/nrc3932 PMC4859809

[2] Karube Y, Tanaka H, Osada H, Tomida S, Tatematsu Y, Yanagisawa K, Yatabe Y, Takamizawa J, Miyoshi S, Mitsudomi T, Takahashi T 2005 Reduced expression of Dicer associated with poor prognosis in lung cancer patients. Cancer Sci 96:111–115 15723655 10.1111/j.1349-7006.2005.00015.x PMC11158408

[3] Ramírez-Moya J, Wert-Lamas L, Riesco-Eizaguirre G, Santisteban P 2019 Impaired microRNA processing by DICER1 downregulation endows thyroid cancer with increased aggressiveness. Oncogene 38:5486–5499 30967628 10.1038/s41388-019-0804-8 PMC6755984

[4] Lin RJ, Lin YC, Chen J, Kuo HH, Chen YY, Diccianni MB, London WB, Chang CH, Yu AL 2010 microRNA signature and expression of Dicer and Drosha can predict prognosis and delineate risk groups in neuroblastoma. Cancer Res 70:7841–7850 20805302 10.1158/0008-5472.CAN-10-0970 PMC4095771

[5] Merritt WM, Lin YG, Han LY, Kamat AA, Spannuth WA, Schmandt R, Urbauer D, Pennacchio LA, Cheng JF, Nick AM, Deavers MT, Mourad-Zeidan A, Wang H, Mueller P, Lenburg ME, Gray JW, Mok S, Birrer MJ, Lopez-Berestein G, Coleman RL, Bar-Eli M, Sood AK 2008 Dicer, Drosha, and outcomes in patients with ovarian cancer. N Engl J Med 359:2641–2650 19092150 10.1056/NEJMoa0803785 PMC2710981

[6] Roman BR, Morris LG, Davies L 2017 The thyroid cancer epidemic, 2017 perspective. Curr Opin Endocrinol Diabetes Obes 24:332–336 28692457 10.1097/MED.0000000000000359 PMC5864110

[7] Somnay YR, Yu XM, Lloyd RV, Leverson G, Aburjania Z, Jang S, Jaskula-Sztul R, Chen H 2017 Notch3 expression correlates with thyroid cancer differentiation, induces apoptosis, and predicts disease prognosis. Cancer 123:769–782 27861750 10.1002/cncr.30403 PMC5319883

[8] Costamagna E, García B, Santisteban P 2004 The functional interaction between the paired domain transcription factor Pax8 and Smad3 is involved in transforming growth factor-beta repression of the sodium/iodide symporter gene. J Biol Chem 279:3439–3446 14623893 10.1074/jbc.M307138200

[9] López-Márquez A, Fernández-Méndez C, Recacha P, Santisteban P 2019 Regulation of Foxe1 by thyrotropin and transforming growth factor beta depends on the interplay between thyroid-specific, CREB and SMAD transcription factors. Thyroid 29:714–725 30652527 10.1089/thy.2018.0136

[10] Vassart G, Dumont JE 1992 The thyrotropin receptor and the regulation of thyrocyte function and growth. Endocr Rev 13:596–611 1425489 10.1210/edrv-13-3-596

[11] Bruno R, Ferretti E, Tosi E, Arturi F, Giannasio P, Mattei T, Scipioni A, Presta I, Morisi R, Gulino A, Filetti S, Russo D 2005 Modulation of thyroid-specific gene expression in normal and nodular human thyroid tissues from adults: an in vivo effect of thyrotropin. J Clin Endocrinol Metab 90:5692–5697 16076943 10.1210/jc.2005-0800

[12] De Felice M, Di Lauro R 2004 Thyroid development and its disorders: genetics and molecular mechanisms. Endocr Rev 25:722–746 15466939 10.1210/er.2003-0028

[13] Fernández LP, López-Márquez A, Santisteban P 2015 Thyroid transcription factors in development, differentiation and disease. Nat Rev Endocrinol 11:29–42 25350068 10.1038/nrendo.2014.186

[14] Lu C, Zhao L, Ying H, Willingham MC, Cheng SY 2010 Growth activation alone is not sufficient to cause metastatic thyroid cancer in a mouse model of follicular thyroid carcinoma. Endocrinology 151:1929–1939 20133453 10.1210/en.2009-1017 PMC2851190

[15] Liu T, Men Q, Su X, Chen W, Zou L, Li Q, Song M, Ouyang D, Chen Y, Li Z, Fu X, Yang A 2017 Downregulated expression of TSHR is associated with distant metastasis in thyroid cancer. Oncol Lett 14:7506–7512 29344196 10.3892/ol.2017.7122 PMC5755142

[16] Davies T, Marians R, Latif R 2002 The TSH receptor reveals itself. J Clin Invest 110:161–164 12122107 10.1172/JCI16234 PMC151075

[17] Rodriguez W, Jin L, Janssens V, Pierreux C, Hick AC, Urizar E, Costagliola S 2012 Deletion of the RNaseIII enzyme dicer in thyroid follicular cells causes hypothyroidism with signs of neoplastic alterations. PLoS One 7:e29929 22242190 10.1371/journal.pone.0029929 PMC3252359

[18] Frezzetti D, Reale C, Calì G, Nitsch L, Fagman H, Nilsson O, Scarfò M, De Vita G, Di Lauro R 2011 The microRNA-processing enzyme Dicer is essential for thyroid function. PLoS One 6:e27648 22132122 10.1371/journal.pone.0027648 PMC3221669

[19] Riesco-Eizaguirre G, Wert-Lamas L, Perales-Paton J, Sastre-Perona A, Fernandez LP, Santisteban P 2015 The miR-146b-3p/PAX8/NIS regulatory circuit modulates the differentiation phenotype and function of thyroid cells during carcinogenesis. Cancer Res 75:4119–4130 26282166 10.1158/0008-5472.CAN-14-3547

[20] Chen CA, Okayama H 1988 Calcium phosphate-mediated gene transfer: a highly efficient transfection system for stably transforming cells with plasmid DNA. Biotechniques 6:632–638 3273409

[21] De la Vieja A, Ginter CS, Carrasco N 2005 Molecular analysis of a congenital iodide transport defect: G543E impairs maturation and trafficking of the Na+/I- symporter. Mol Endocrinol 19:2847–2858 15976004 10.1210/me.2005-0162

[22] Kimura T, Van Keymeulen A, Golstein J, Fusco A, Dumont JE, Roger PP 2001 Regulation of thyroid cell proliferation by TSH and other factors: a critical evaluation of in vitro models. Endocr Rev 22:631–656 11588145 10.1210/edrv.22.5.0444

[23] Pasca di Magliano M, Di Lauro R, Zannini M 2000 Pax8 has a key role in thyroid cell differentiation. Proc Natl Acad Sci U S A 97:13144–13149 11069301 10.1073/pnas.240336397 PMC27192

[24] De la Vieja A, Santisteban P 2018 Role of iodide metabolism in physiology and cancer. Endocr Relat Cancer 25:R225–R245 29437784 10.1530/ERC-17-0515

[25] Ravera S, Reyna-Neyra A, Ferrandino G, Amzel LM, Carrasco N 2017 The Sodium/Iodide Symporter (NIS): molecular physiology and preclinical and clinical applications. Annu Rev Physiol 79:261–289 28192058 10.1146/annurev-physiol-022516-034125 PMC5739519

[26] Rowe CW, Paul JW, Gedye C, Tolosa JM, Bendinelli C, McGrath S, Smith R 2017 Targeting the TSH receptor in thyroid cancer. Endocr Relat Cancer 24:R191–R202 28351942 10.1530/ERC-17-0010

[27] Choudhury PS, Gupta M 2018 Differentiated thyroid cancer theranostics: radioiodine and beyond. Br J Radiol 91:20180136 30260232 10.1259/bjr.20180136 PMC6475953

[28] Fabbro D, Di Loreto C, Beltrami CA, Belfiore A, Di Lauro R, Damante G 1994 Expression of thyroid-specific transcription factors TTF-1 and PAX-8 in human thyroid neoplasms. Cancer Res 54:4744–4749 8062273

[29] Ros P, Rossi DL, Acebrón A, Santisteban P 1999 Thyroid-specific gene expression in the multi-step process of thyroid carcinogenesis. Biochimie 81:389–396 10401674 10.1016/s0300-9084(99)80086-8

[30] Zhang P, Zuo H, Nakamura Y, Nakamura M, Wakasa T, Kakudo K 2006 Immunohistochemical analysis of thyroid-specific transcription factors in thyroid tumors. Pathol Int 56:240–245 16669872 10.1111/j.1440-1827.2006.01959.x

[31] Kimura S 2011 Thyroid-specific transcription factors and their roles in thyroid cancer. J Thyroid Res 2011:710213 21687604 10.4061/2011/710213 PMC3112524

[32] Network CGAR 2014 Integrated genomic characterization of papillary thyroid carcinoma. Cell 159:676–690 25417114 10.1016/j.cell.2014.09.050 PMC4243044

[33] Ruiz-Llorente S, Carrillo Santa de Pau E, Sastre-Perona A, Montero-Conde C, Gómez-López G, Fagin JA, Valencia A, Pisano DG, Santisteban P 2012 Genome-wide analysis of Pax8 binding provides new insights into thyroid functions. BMC Genomics 13:147 22531031 10.1186/1471-2164-13-147 PMC3403905

[34] Di Palma T, Nitsch R, Mascia A, Nitsch L, Di Lauro R, Zannini M 2003 The paired domain-containing factor Pax8 and the homeodomain-containing factor TTF-1 directly interact and synergistically activate transcription. J Biol Chem 278:3395–3402 12441357 10.1074/jbc.M205977200

[35] Taki K, Kogai T, Kanamoto Y, Hershman JM, Brent GA 2002 A thyroid-specific far-upstream enhancer in the human sodium/iodide symporter gene requires Pax-8 binding and cyclic adenosine 3',5'-monophosphate response element-like sequence binding proteins for full activity and is differentially regulated in normal and thyroid cancer cells. Mol Endocrinol 16:2266–2282 12351692 10.1210/me.2002-0109

[36] Chun JT, Di Dato V, D'Andrea B, Zannini M, Di Lauro R 2004 The CRE-like element inside the 5'-upstream region of the rat sodium/iodide symporter gene interacts with diverse classes of b-Zip molecules that regulate transcriptional activities through strong synergy with Pax-8. Mol Endocrinol 18:2817–2829 15319451 10.1210/me.2004-0020

