# Supplementary material for: A Positive Feedback Loop Between DICER1 and Differentiation Transcription Factors Is Important for Thyroid Tumorigenesis
Source: Thyroid. 2021 Jun 8;31(6):912–21. doi: 10.1089/thy.2020.0297 (PMC8215414; doi:10.1089/thy.2020.0297)
Supplement: Supplemental data [file Supp_Data.docx]

**Supplementary Material and Methods.**

*Cell culture*

The following cell lines were used: rat PCCl3 cells, provided by Dr. A. Fusco (Istituto di Endocrinologia ed Oncologia Sperimentale-CNR, Dipartimento di Medicina Molecolare e Biotecnologie Mediche, Università degli Studi di Napoli "Federico II," Naples, Italy); human thyroid cancer cells (i) Cal62, obtained from Leibniz-Institut DSMZ-German Collection of Microorganisms and Cell Cultures, (ii) TPC1, provided by Dr. A.P. Dackiw (Johns Hopkins University, Baltimore, MD), (iii) SW1736, provided by Dr. N.E. Heldin (University of Uppsala, Uppsala, Sweden) and (iv) BCPAP, provided by Dr. M Santoto (Università degli Studi di Napoli "Federico II," Naples, Italy). Human cervical cancer cells (HeLa) were from the American Type Culture Collection, (Manassas, VA). All cell lines were authenticated every 6 months by short tandem repeat profiling using the Applied Biosystems Identifier kit at the Genomic Facility of the Instituto de Investigaciones Biomédicas (Madrid, Spain).

Rat normal PCCl3 thyroid cells (1) were cultured in Coon’s modified Ham’s F-12 medium supplemented with 5% donor calf serum, glutamine, antibiotics, and a six-hormone medium mixture containing the following: 1 nM bovine TSH, 10 μg/mL insulin, 10 ng/mL somatostatin, 10 μg/mL transferrin, 10 nM hydrocortisone, and 10 ng/mL glycyl-L-histidyl-L-lysine acetate. The effects of TSH were studied by starving nearly confluent PCCl3 cells of TSH, insulin and serum (0.1%) for two days (medium 4H) before adding 1 nM TSH or 10 μM forskolin (both from Sigma-Aldrich, Madrid, Spain) to the culture medium. In some experiments, the PKA inhibitor H89 (Sigma-Aldrich) was added to the medium one hour before hormone addition at a final concentration of 10 μM.

Human thyroid cancer cell lines representative of PTC (TPC1 and BCPAP) and ATC (Cal62 and SW1736), and the HeLa cell line, were cultured in Dulbecco’s modified Eagle’s medium (DMEM) supplemented with 10% fetal bovine serum, glutamine, antibiotics and sodium pyruvate.

*Transfections, plasmids, constructs, and siRNAs*

The DharmaFECT transfection reagent (Dharmacon GE Healthcare, Lafayette, CO) was used to transfect *DICER1* siRNAs into PCCl3 cells, Lipofectamine 2000 (Thermo Fisher Scientific, Waltham, MA) was used to transfect the thyroid cancer cell lines, and calcium chloride was used to transfect HeLa cells (2).

The *DICER1* expression vector was kindly provided by Dr. Richard Gregory (Boston Children’s Hospital) (3), and the pGL3-DICER-Prom vector was a gift from Dr. David Fisher (Addgene, plasmid #25851) (4). Serial deletion mutants of the DICER1 promoter were subcloned in the pGL3 vector using the restriction enzymes HindIII and KpnI. To do this, DNA fragments from the DICER1 promoter were amplified from the pGL3-DICER-Prom vector by PCR using the primers described in the Supplementaty Table I (purchased from Sigma-Aldrich). Purified products were then digested with the aforementioned restriction enzymes and subcloned into the pGL3-Luc vector. The fidelity of the amplified DNA was verified by Sanger sequencing at the Genomic Facilities of Instituto de Investigaciones Biomédicas. NKX2-1, CREB, CREM and PAX8 expression vectors have been previously described (5). DICER1 siRNAs were purchased from Thermo Fisher Scientific (Waltham, MA; Silencer® Select Pre-Designed siRNA Dicer1 s23754 and s23755). CREB was silenced using ON-TARGET plus SMARTpool siRNA molecules (Dharmacon).

*RNA quantification*

RNA was extracted using TRIzol reagent (Thermo Fisher Scientific) and equal amounts were added to a reverse-transcriptase (RT) reaction mix of the M-MLV Reverse Transcriptase Kit (Promega Corp.). Quantitative RT-PCR was performed on the Mx3000P QPCR platform (Agilent Technologies, Santa Clara, CA) using KAPA Sybr Fast qPCR Master Mix (Merck KGaA, Darmstadt, Germany). Relative levels of the qPCR products were expressed as a function of β-actin expression for human samples or β-glucuronidase (GUS) for PCCl3 samples. All primers were purchased from Sigma-Aldrich and are described in Supplementary Table I.

*Protein extraction and western blotting.*

Cellular proteins were extracted as described (6, 7). Protein concentration was measured by the Bradford method using a kit from Bio-Rad Laboratories (Hercules, CA). Samples were separated by SDS-PAGE and transferred to nitrocellulose membranes (Bio-Rad). Membranes were blocked in phosphate buffered saline (PBS)-T buffer (PBS +0.1% Tween 20, pH 7.5) containing 5% nonfat milk. Antibody binding was revealed with horseradish peroxidase (HRP)-conjugated secondary antibodies and immunoreactive proteins were visualized with the Luminol Western Blot Detection Reagent (Thermo Fisher Scientific). The resultant films were scanned and quantified using Image J (NIH, Bethesda, MD).

The following primary antibodies were employed for analyses: DICER1 A-2 (sc-136981) (used for PCCL3 cell extracts), β-actin (sc-1616R) and vinculin (sc-25336) were purchased from Santa Cruz Biotechnology (Santa Cruz, CA); DICER 13D6 (ab14601) and NKX2-1 (ab76013) (both used for human samples) were purchased from Abcam (Cambridge, UK); and PAX8 (PA0300) and NKX2-1 (PA 0100) (used for PCCL3 cell extracts) were purchased from Biopat (Milan, Italy). The NIS antibody was kindly provided by Dr. Nancy Carrasco (Vandervilt University, Nashville, TN).

*Chromatin immunoprecipitation assay*

Chromatin immunoprecipitation (ChIP) was performed using the ChIP-IT Express Enzymatic kit (Enzymatic shearing kit from Active Motif, Shanghai, China). Cross-linked BCPAP chromatin was immunoprecipitated using a polyclonal antibody against NKX2-1 (ab76013 from Abcam) or Rabbit IgG (Merk Millipore Burlington, MA) as a control. Immunoprecipitated samples were assayed by qPCR using eight specific primers for the analyzed regions on the DICER1 promoter (Supplementary Table I). The predicted binding sites 1512–1528 and 2109–2138 were amplified in the same region because of their proximity. The enrichment of target sequences in ChIP experiments was calculated relative to the negative IgG controls.

*Electrophoretic mobility shift assay*

Oligonucleotides containing a NKX2-1 binding site within the DICER1 promoter were designed (Supplementary Table I), purchased (Sigma-Aldrich) and labeled with 25 µCi [γ^32^P]-ATP using T4 polynucleotide kinase (Promega Corp.). Labeled oligonucleotides were purified on Quick Spin G-25 Sephadex columns (Roche Life Sciences, Mannheim, Germany). Recombinant NKX2-1 protein was obtained using an *in vitro* transcription-translation (TNT) kit (Promega Corp.) and was incubated with the labeled probes, as described (8).

*Iodide transport*

PCCl3 cells were assayed for iodide transport 48 hours after Dicer1 silencing. We used differentiated normal PCCl3 cells for analysis as tumoral cells have lost NIS expression and function. Cells were incubated for 1 hour in PBS containing 20 μM potassium iodide, 2 mM methyl-mercapto-imidazole, and Na125I (specific activity, 100 μCi/mmol I-). Radioactivity was quantified in a γ-counter. Uptake was expressed as counts per minute and normalized to the amount of DNA in each well. Perchlorate (ClO4-), a competitive inhibitor of NIS-mediated iodide transport, was used at 80 μM.

**Supplementary references:**

**1.** Fusco A, Berlingieri MT, Di Fiore PP, Portella G, Grieco M, Vecchio G 1987 One- and two-step transformations of rat thyroid epithelial cells by retroviral oncogenes. Mol Cell Biol **7**:3365-3370.

**2.** Chen CA, Okayama H 1988 Calcium phosphate-mediated gene transfer: a highly efficient transfection system for stably transforming cells with plasmid DNA. Biotechniques **6**:632-638.

**3.** Chendrimada TP, Gregory RI, Kumaraswamy E, Norman J, Cooch N, Nishikura K, Shiekhattar R 2005 TRBP recruits the Dicer complex to Ago2 for microRNA processing and gene silencing. Nature **436**:740-744.

**4.** Levy C, Khaled M, Robinson KC, Veguilla RA, Chen PH, Yokoyama S, Makino E, Lu J, Larue L, Beermann F, Chin L, Bosenberg M, Song JS, Fisher DE 2010 Lineage-specific transcriptional regulation of DICER by MITF in melanocytes. Cell **141**:994-1005.

**5.** López-Márquez A, Fernández-Méndez C, Recacha P, Santisteban P 2019 Regulation of Foxe1 by Thyrotropin and Transforming Growth Factor Beta Depends on the Interplay Between Thyroid-Specific, CREB and SMAD Transcription Factors. Thyroid **29**:714-725.

**6.** Ramírez-Moya J, Wert-Lamas L, Santisteban P 2018 MicroRNA-146b promotes PI3K/AKT pathway hyperactivation and thyroid cancer progression by targeting PTEN. Oncogene **37**:3369-3383.

**7.** Ramírez-Moya J, Wert-Lamas L, Riesco-Eizaguirre G, Santisteban P 2019 Impaired microRNA processing by DICER1 downregulation endows thyroid cancer with increased aggressiveness. Oncogene **38**:5486-5499.

**8.** Leoni SG, Kimura ET, Santisteban P, De la Vieja A 2011 Regulation of thyroid oxidative state by thioredoxin reductase has a crucial role in thyroid responses to iodide excess. Mol Endocrinol **25**:1924-1935.
