# Supplementary material for: A Positive Feedback Loop Between DICER1 and Differentiation Transcription Factors Is Important for Thyroid Tumorigenesis
Source: Thyroid. 2021 Jun 8;31(6):912–21. doi: 10.1089/thy.2020.0297 (PMC8215414; doi:10.1089/thy.2020.0297)
Supplement: Supplemental data [file Supp_TableS1.docx]

| **GENE** | **FORWARD OLIGO** | **REVERSE OLIGO** |
| --- | --- | --- |
| DICER1 | GGTGGTCCACGAGTCACAAT | TAGCACTGCCTTCGTTTCGT |
| ACTIN | CACTCTTCCAGCCTTCCTT | CTCGTCATACTCCTGCTTGCT |
| NKX2.1 | GCAACGGCAACCTGGGCAAC | ATGAAGCGGGAGATGGCGGG |
| PAX8 | CAAGGTGGTGGAGAAGATTG | GAGGTTGAATGGTTGCTG |
| GUS | CATGACGAACCAGTCACCAC | ACGGTCTGCTTCCCATACAC |
| NKX2-1 binding site (EMSA) | TGACAGATTGCAAGAGGGCAACCA | TGGTTGCCCTCTTGCAATCTGTCA |

**SUPPL. TABLE I**. Oligonucleotides

qPCR and EMSA primers:

ChIP primers (qPCR):

| **PREDICTED NKX2-1 SITE** | **FORWARD OLIGO** | **REVERSE OLIGO** |
| --- | --- | --- |
| -102 | TCACAGGCTCGCTCTCATGC | CACGGCCCGCGGCAAC |
| -308 | TGCCAAACTTAGCCGGCCTC | CACTCAGGAGCAGGGAGACAG |
| -696 | CCACTGAAGAGATGTCTACATTTGG | TGGCCCTCTGTTTGTAAGCTG |
| -1159 | AGCACCTACCTCACAGGGTG | AAATGTCACCTGTGGCCTGC |
| -1512 and -1528 | ACAGGAAGCAGGCTGGGTAC | GAAGAGACGGGGTTTCACCATG |
| -1751 | ATGGGTCTGCCAGGAGCATC | TGAGGCTGGTGTGCTCAGTC |
| -2109 and -2138 | AATCAGGTATACCTGCTGATGG | GTGGGCTGGTACAACTTCAG |
| -2224 | GAGATTGCGCCATTGCAC | GTTCCATCAGCAGGTATACCTG |

Cloning primers:

| **CONSTRUCT** | **FORWARD OLIGO** | **REVERSE OLIGO** |
| --- | --- | --- |
| -1 to -625 | TAAGCAGGTACCCCAAATGCGGAGGCCCCT | TGCTTAAAGCTTTCGTCCCCGCTGTCAGGTTA |
| -1 to -1250 | TAAGCAGGTACCCTACCTCACAGGGTGCAC | TGCTTAAAGCTTTCGTCCCCGCTGTCAGGTTA |
| -1 to -1866 | TAAGCAGGTACCAATACATGGGTCTGCCAGGA | TGCTTAAAGCTTTCGTCCCCGCTGTCAGGTTA |
