# Supplementary material for: A Positive Feedback Loop Between DICER1 and Differentiation Transcription Factors Is Important for Thyroid Tumorigenesis
Source: Thyroid. 2021 Jun 8;31(6):912–21. doi: 10.1089/thy.2020.0297 (PMC8215414; doi:10.1089/thy.2020.0297)
Supplement: Supplemental data [file Supp_FigureS1.docx]

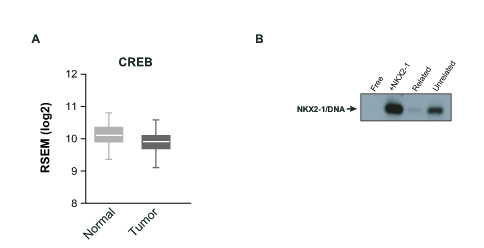


**FIG S1.** **(A)** CREB levels in normal and tumor samples obtained from TCGA data

**(B)** Electrophoretic mobility shift assay to assess NKX2-1 binding to the DICER1 promoter. The arrow marks the primary DNA-NKX2-1 complex. The binding specificity was performed using related or unrelated oligonucleotides.
