# Supplementary material for: A Positive Feedback Loop Between DICER1 and Differentiation Transcription Factors Is Important for Thyroid Tumorigenesis
Source: Thyroid. 2021 Jun 8;31(6):912–21. doi: 10.1089/thy.2020.0297 (PMC8215414; doi:10.1089/thy.2020.0297)
Supplement: Supplemental data [file Supp_FigureS2.docx]

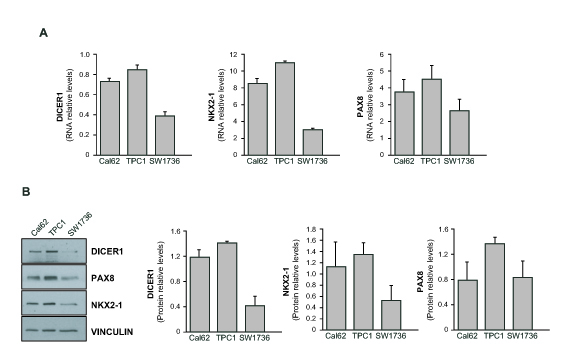


**FIG S2.** DICER1, NKX2-1 and PAX8 mRNA **(A)** and protein **(B)** levels in the thyroid cancer cell lines Cal62, TPC1 and SW1736. Vinculin was used as loading control in the immunoblots.
